# Emerging Mechanisms of G_1_/S Cell Cycle Control by Human and Mouse Cytomegaloviruses

**DOI:** 10.1128/mBio.02934-21

**Published:** 2021-12-14

**Authors:** Boris Bogdanow, Quang Vinh Phan, Lüder Wiebusch

**Affiliations:** a Department of Structural Biology, Leibniz-Forschungsinstitut für Molekulare Pharmakologie, Berlin, Germany; b Department of Pediatrics, Division of Oncology and Hematology, Charité - Universitätsmedizin Berlin, Berlin, Germany; University of Wisconsin—Madison; Albert Einstein College of Medicine

**Keywords:** DNA replication, anaphase-promoting complex, cell cycle, cell cycle checkpoints, cyclins, cytomegalovirus, tumor suppressor genes

## Abstract

Cytomegaloviruses (CMVs) are among the largest pathogenic viruses in mammals. To enable replication of their long double-stranded DNA genomes, CMVs induce profound changes in cell cycle regulation. A hallmark of CMV cell cycle control is the establishment of an unusual cell cycle arrest at the G_1_/S transition, which is characterized by the coexistence of cell cycle stimulatory and inhibitory activities. While CMVs interfere with cellular DNA synthesis and cell division, they activate S-phase-specific gene expression and nucleotide metabolism. This is facilitated by a set of CMV gene products that target master regulators of G_1_/S progression such as cyclin E and A kinases, Rb-E2F transcription factors, p53-p21 checkpoint proteins, the APC/C ubiquitin ligase, and the nucleotide hydrolase SAMHD1. While the major themes of cell cycle regulation are well conserved between human and murine CMVs (HCMV and MCMV), there are considerable differences at the level of viral cell cycle effectors and their mechanisms of action. Furthermore, both viruses have evolved unique mechanisms to sense the host cell cycle state and modulate the infection program accordingly. This review provides an overview of conserved and divergent features of G_1_/S control by MCMV and HCMV.

## INTRODUCTION

*Omnis cellula e cellula* is the universal principle of reproduction and growth in living organisms (1). The underlying molecular processes are orchestrated by the cell division cycle (2). The cell cycle comprises a highly regulated series of events required for faithful duplication of cellular components and their segregation into two daughter cells. The basic mechanisms of cell cycle control are conserved from yeast to humans. The four major cell cycle phases are mitosis (M), gap 1 (G_1_), DNA synthesis (S), and gap 2 (G2). Nonproliferating cells withdraw from the cell cycle in early G_1_ and can reach a quiescent “G_0_” state. Upon growth factor stimulation, cells pass a “restriction point” in late G_1_, and then cell cycle progression becomes independent from extracellular signals. Consequently, cells at the G_1_/S transition are committed to DNA synthesis and cell division, unless the activation of DNA damage checkpoints stops the cell cycle machinery to prevent mitosis and allow time for DNA repair. A dysfunctional cell cycle control can cause genetic instability and cancer (3, 4).

Cell cycle regulation is also a central aspect of virus biology (5). As obligate intracellular parasites, viruses rely on a multitude of cellular activities and supplies that are under strict control of the cell cycle. The majority of cells in the human body are noncycling and contain very low levels of nucleotides and replication factors, thus limiting virus replication (6). To overcome these restrictions and create a favorable metabolic state, viruses have evolved powerful cell cycle regulators, targeting the most crucial checkpoints of G_0_/G_1_/S transition. Studying these viral regulators has aided tremendously in revealing the major principles of cell cycle regulation. In particular, research on small DNA tumor viruses led to the discovery of p53 (TP53), Rb (RB1), and E2F1, which are master regulators of the G_1_/S cell cycle transition (7, 8).

Due to their limited coding capacity, small DNA viruses depend on host enzymes for DNA replication and therefore have a vital interest in pushing infected cells from G_0_/G_1_ into S phase. In contrast, large DNA viruses such as herpesviruses encode their own replication apparatus, which makes them independent from cellular DNA polymerases. Active host DNA synthesis may even hinder efficient herpesvirus replication by competing for limited pools of nucleotides and other replication factors. Accordingly, a number of herpesviruses have evolved mechanisms to inhibit S-phase entry of productively infected cells (9). However, like other viruses, herpesviruses require a metabolic state and gene expression pattern that is characteristic for replicating cells. Therefore, herpesviruses express a mixture of cell cycle stimulating and inhibiting activities. In addition, herpesvirus gene products modulate the cell cycle in a way that allows maintenance and propagation of viral genomes in latently infected cells. This flexible adjustment to different infection modes is facilitated by a diverse set of cell cycle regulators, including viral orthologs of cyclins and cyclin-dependent kinases (CDKs) (10, 11).

Cytomegaloviruses (CMVs) form a subgroup of betaherpesviruses that is characterized by a pronounced species specificity, a broad cell tropism, and a prolonged replication cycle of up to several days. Due to the latter property, CMVs particularly rely on a stable cell cycle environment that is not interrupted by chromatin condensation or cell division. In order to create and maintain such a cell cycle state near the G_1_/S border, CMVs employ an array of G_1_/S control functions. Over recent years a number of novel human and murine CMV-encoded cell cycle regulators have been identified. This was facilitated by important technological advances: first, the cloning of CMV genomes as bacterial artificial chromosomes made CMVs accessible for reverse genetics (12); second, the development of genetic recombineering methods allowed the precise and efficient mutagenesis of these cloned CMV genomes (13); and third, the interactome analysis of CMV gene products by affinity purification-mass spectrometry enabled the sensitive and systematic detection of novel cell cycle binding partners (14). Here, we review recent developments and emerging mechanisms of cell cycle control by murine and human CMVs with emphasis on the G_1_/S transition.

## CMV EFFECTORS DISTURBING CELLULAR MECHANISMS OF G_1_/S CONTROL

**FIG 1:**
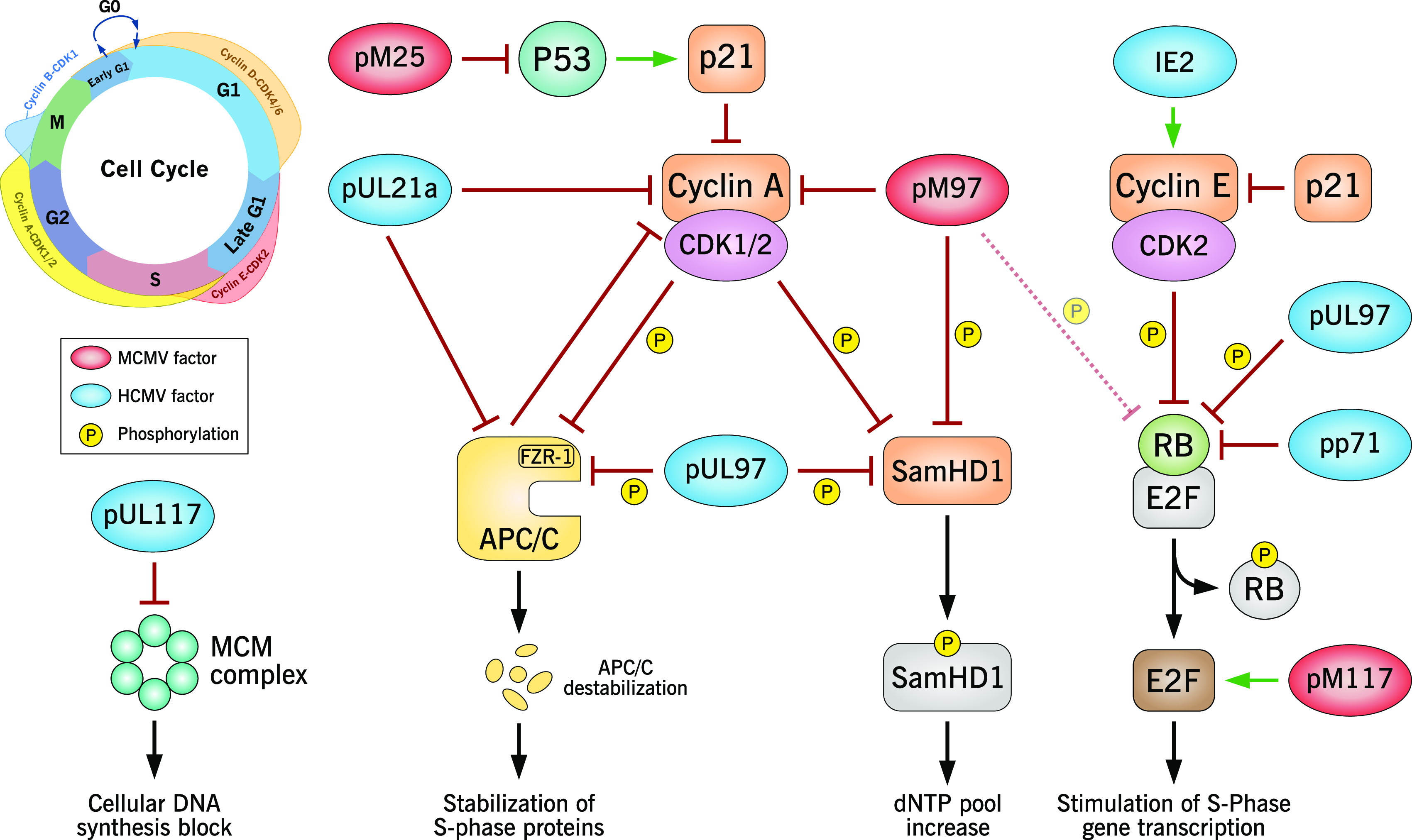
Schematic overview of G_1_/S cell cycle subversion strategies used by CMVs. Cell cycle effectors of HCMV (highlighted in blue) and MCMV (highlighted in red) target key factors in the regulatory network governing G_1_/S transition. This results in a dissociation of downstream cell cycle activities, where viral stimulation of S-phase gene expression and nucleotide metabolism is accompanied by viral inhibition of cellular DNA synthesis. Phosphorylations are indicated by an encircled “P”; the inhibition or destabilization of host factors is represented by red lines with bar heads.

Major molecular processes of G_1_/S transition manipulated by CMVs are cellular DNA synthesis, E2F-dependent gene transcription, anaphase promoting complex/cyclosome (APC/C)-dependent protein degradation and cellular deoxyribonucleotide triphosphate (dNTP) metabolism. Each of these processes is specifically targeted by CMV gene products, as reviewed in detail in the following subsections. The different levels of G_1_/S cell cycle control as well as the CMV cell cycle regulators and their specific points of attack are schematically summarized in Fig. 1.

### Dissociation of cyclins A and E, the drivers of G_1_/S transition.

The major drivers of G_1_/S transition are cyclin A (CCNA2) and cyclin E (CCNE1). Both share a catalytic subunit, CDK2, and a considerable number of interactors (15) and substrates (16). However, their abundance levels are differently regulated across the cell cycle, and their substrate specificities are sufficiently divergent to ensure different functional outputs. The interaction of human and murine CMVs with cyclins A and E is highly adapted to the distinct roles these cyclins play at the G_1_/S transition, with the consequence that cyclin E is strongly activated by both viruses, whereas cyclin A is potently inhibited (17–20). The dichotomous regulation of G_1_/S cyclins is the most efficient way to create favorable conditions for virus growth since it enables manipulation of cell cycle-associated signaling from the most upstream stage, i.e., before the ensuing phosphorylation-dependent processes are kicked off.

Cyclin E controls activities conducive to a replicating DNA virus, such as the expression of genes controlled by E2F-type transcription factors (21) and the production of enzymes required for DNA synthesis (22). Consistently, both murine (19) and human (18, 23, 24) CMVs upregulate the expression and activity of cyclin E. In the case of HCMV, cyclin E expression is activated at the transcriptional level by the 86-kDa immediate early-2 (IE2; pUL122) protein which recognizes a cyclin E promoter sequence matching the IE2 binding site consensus (23). IE2 shares an N-terminal transactivation domain with the second major immediate-early gene product of HCMV, the 72-kDa immediate early-1 protein (IE1; pUL123). A recombinant HCMV, bearing a deletion of amino acids 30 to 77 within this shared N terminus, is unable to induce cyclin E expression, even when intact full-length IE1 is complemented (25). This finding supports the importance of IE2 for cyclin E expression in the context of HCMV infection. In noninfected cells, cyclin E transcription requires cyclin D-associated kinase activity, which acts upstream of cyclin E (26). Thus, the kickstart of cyclin E transcription by IE2 makes HCMV independent of cyclin D (27) and allows for a rapid increase of cyclin E activity after the onset of viral gene expression. Of note, at late times of infection, cyclin E transcript levels are still high but unresponsive to targeted degradation of IE2 (28). Since cyclin E is an E2F-responsive gene (29), it appears likely that E2F activation by viral UL82 and UL97 gene products (see next section) can establish an IE2-independent mode of cyclin E transcription during the course of HCMV infection. Whether cyclin E activation is responsible for the general induction of E2F-responsive genes in IE2-expressing cells (30) or whether this is a more direct effect of a yet poorly understood IE2-Rb interaction (31, 32) is unclear.

IE1 also contributes to activation of cyclin E-CDK2 by acting at the protein level. The interaction of IE1 with the p107 (RBL1) N-terminal domain, releases cyclin E from the inhibitory action of p107 (33, 34). For MCMV, the molecular details of viral cyclin E activation are yet unknown. However, the high degree of conservation between IE1/IE2 and their MCMV homologs ie1/ie3 (M123/M122) (35), as well as the early timing of cyclin E induction in MCMV-infected cells (19), suggests that these MCMV gene products play a comparable role in the control of cyclin E activity.

Active cyclin E in cycling, noninfected cells is highly unstable due to a potent negative autoregulatory feedback loop that is based on SCF-Fbxw7-mediated ubiquitylation of autophosphorylated cyclin E (36). The resulting proteasomal degradation of cyclin E explains the sharp decrease of cyclin E-CDK2 activity after G_1_/S transition. In contrast, CMV-infected cells display high levels of cyclin E protein and kinase activity for prolonged periods of time, resembling the aberrant cyclin E overexpression in cancer cells (37). Although the underlying mechanisms of cyclin E protein stabilization in infected cells are yet unexplored, the increase in active serine/threonine phosphatases PP1 and PP2A during HCMV infection (38) may protect cyclin E from phosphodegron phosphorylation (39, 40).

**FIG 2:**
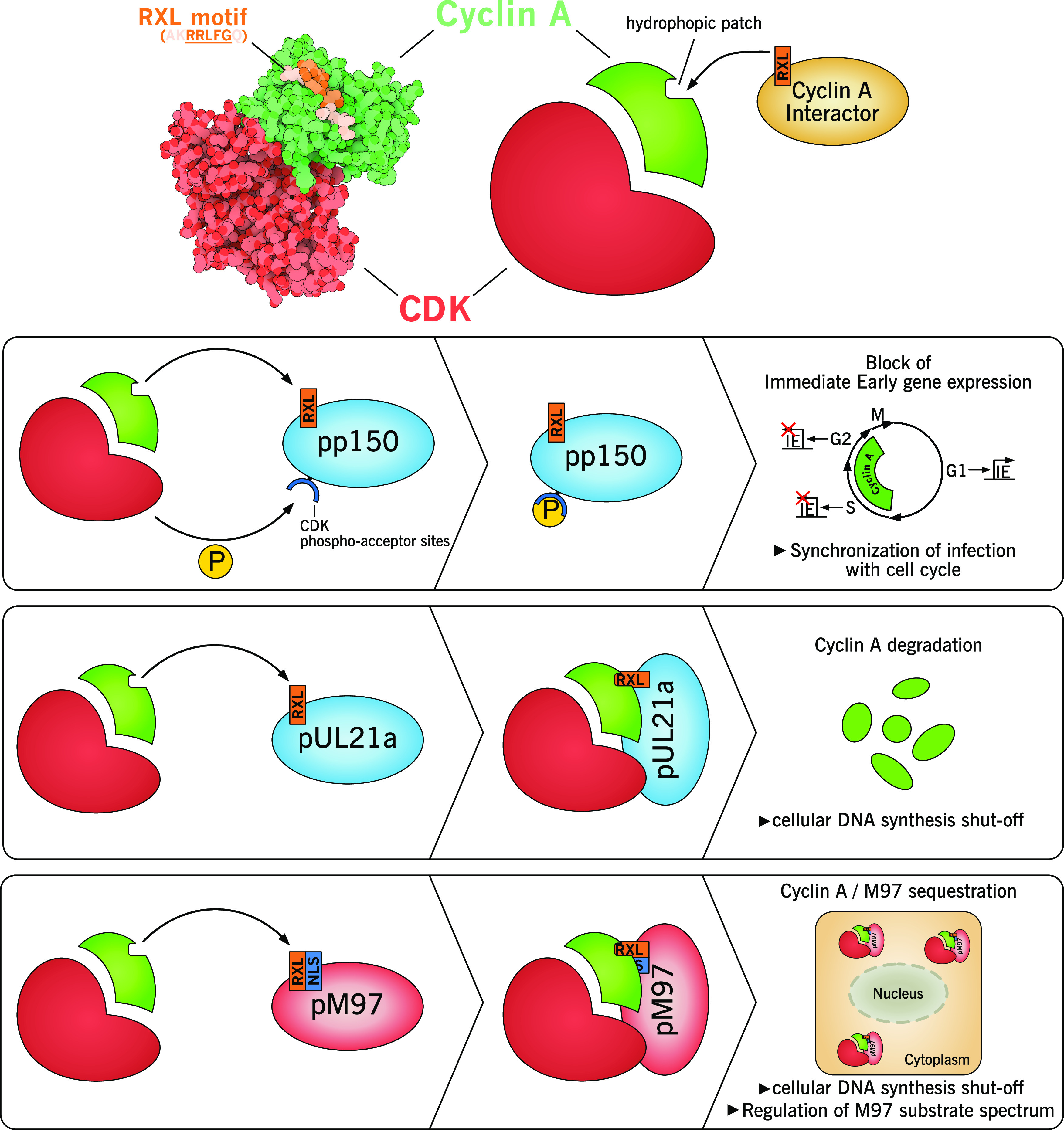
Mechanisms of cyclin A control by CMV-encoded proteins. As the critical regulator of S phase, cyclin A is targeted by several CMV factors for degradation, phosphorylation, or sequestration. All factors exploit an RxL sequence motif for tight and specific binding to a hydrophobic patch in the cyclin A structure. Upon cyclin A binding, the HCMV tegument protein pp150 is phosphorylated by the CDK subunit, resulting in a block of viral gene expression in S/G_2_ cell cycle phases. The interaction with HCMV-pUL21a leads to proteasomal degradation of cyclin A and, in consequence, to inhibition of cellular DNA replication. Complex formation of the MCMV kinase M97 with cyclin A-CDK precludes both kinases from nuclear entry and therefore has two functional outcomes: the shutoff of cellular DNA synthesis and an altered substrate spectrum of M97. For structural rendering of cyclin A-CDK bound to an RXL peptide (top panel), the protein data bank file PDB 1H28 was used.

In contrast to cyclin E, cyclin A activity is accompanied by rather undesired cellular consequences for CMV replication. Cyclin A-CDK2 is crucial for the commitment of late-G_1_ cells to active cellular DNA replication and cyclin A-CDK1 for entry into mitosis (41). To avoid any disturbance of the viral infectious cycle, both HCMV and MCMV efficiently interfere with cyclin A activity via effector proteins that target cyclin A by direct protein-protein interaction (Fig. 2). These interactions are mediated by short linear RxL motifs within the viral protein sequences. Such motifs are well known as cyclin A docking sites in cellular CDK substrates and inhibitors (42). HCMV encodes the small and highly unstable early protein pUL21a that targets cyclin A for proteasomal degradation (43, 44). This is the essential step in viral inhibition of cellular DNA synthesis, as shown by introduction of UL21a-RxL point mutations into the HCMV genome (43, 44). The loss of the G_1_/S cell cycle arrest function also allows infected cells to enter mitosis, which is associated with deleterious consequences, ranging from impaired viral DNA synthesis, irregular metaphase spindle formation, precocious sister chromatid formation, fragmentation of genetic material, and mitotic cell death (44). Importantly, pUL21a exists only in primate CMVs, and there is no MCMV homolog.

MCMV therefore relies on a different strategy of cyclin A inhibition. Here, the viral kinase M97 interacts with cyclin A via an RxL motif in its N-terminal noncatalytic domain. This interaction leads to cytosolic sequestration of cyclin A-M97-CDK complexes (20). Even though cyclin A-M97-CDK complex formation does not interfere with cyclin A-associated kinase activity *per se* (19, 20), it causes viral inhibition of cellular DNA synthesis. That is explained on the one hand by the fact that nuclear localization of cyclin A is required for phosphorylation-dependent activation of cellular DNA replication (45, 46). On the other hand, M97 outcompetes RxL-containing cellular substrates for cyclin A binding. In contrast to HCMV, the loss of G_1_/S arrest function in M97-RxL mutant-infected cells does not result in mitotic entry and cell death but instead in cell accumulation in G_2_ phase (20). This points toward a yet-unidentified mechanism of G_2_ arrest in MCMV-infected cells (47). Interestingly, M97 integrates the antipodal activities of a CDK inhibitor (CKI) with the function of a CDK-like kinase that activates S-phase metabolism. This is in contrast to HCMV, which has dissociated both activities onto two gene products, the viral CDK-like kinase pUL97 and the CKI-like protein pUL21a.

### Activation of S-phase transcription by viral Rb-E2F interactors.

At the level of transcriptional regulation, the most important targets of cyclin-CDK activity are members of the retinoblastoma (Rb) protein family, including the Rb tumor suppressor (RB1) (48) and the two Rb-like pocket proteins p107 (RBL1) and p130 (RBL2). Central to Rb function is a phosphodependent interaction with E2F-DP transcription factors. In G_0_, unphosphorylated p107/p130 binds to E2F4/5-DP1/2 and MuvB to form transcriptionally repressive DREAM complexes (49). In early G_1_, cyclin D-CDK4/6 phosphorylation results in 14 monophosphorylated Rb isoforms that regulate Rb’s preferential binding to different E2Fs and other targets and induce a diverse set of transcriptional responses (26, 50). In late G_1_ phase, Rb multisite phosphorylation by cyclin E-CDK2 leads to the dissociation of Rb-E2F1/2/3 complexes and broad stimulation of S-phase gene transcription (51). Altogether, this puts the Rb protein family in a crucial position as transcription regulators of the G_0_/G_1_/S transition. The importance of Rb-E2F regulation for HCMV and MCMV replication is underpinned by their ability to target Rb-E2F proteins at multiple levels.

HCMV encodes two proteins containing short linear LxCxE motifs that enable interaction with the conserved pocket domains of the Rb protein family. The first is the 71-kDa phosphoprotein (pp71, pUL82), a major constituent of the virus tegument and important antagonist of intrinsic immunity (52). Binding to pp71-LxCxE leads to proteasomal degradation of the unphosphorylated and monophosphorylated forms of Rb and Rb-like proteins (53, 54). This stimulates the cell cycle reentry of quiescent cells and their transition from G_1_ to S phase (53, 55). In contrast to Rb inhibition by LxCxE-containing oncoproteins of DNA tumor viruses (56, 57), pp71 induces neither cellular transformation nor apoptosis (53).

The second HCMV gene product employing an LxCxE motif to regulate the Rb protein family is the viral kinase pUL97 (58, 59). LxCxE-dependent recruitment to the N-terminal noncatalytic domain of pUL97 results in multisite phosphorylation of Rb (59, 60), p107, and p130 (61). As a consequence, Rb and a fraction of p107 and p130 proteins dissociate from E2F target genes, leading to their transcriptional activation (60, 61). IE1 represents a third factor that directly interacts with pocket proteins during HCMV infection but uses an LxCxE-independent mode of binding (62). IE1 binding to p107/p130-containing complexes contributes to the derepression of E2F-responsive promoters (61, 63). Interestingly, both pp71-LxCxE and UL97-LxCxE single-mutant viruses display comparable kinetics of virus growth with wild-type virus (64, 65), suggesting that pp71, pUL97, and IE proteins possibly complement each other in Rb-E2F regulation.

Despite encoding at least three antagonists of pocket protein function, the relationship between HCMV and the Rb family is more complex than originally anticipated. pUL97-dependent phosphorylation leads to increased Rb protein stability and Rb knockdown results in less efficient HCMV replication (66). HCMV does not disrupt the DREAM complex p130-E2F4-MuvB (61) but modulates its activity by pUL97-mediated phosphorylation of the MuvB subunit LIN52 (67). This suggests a model where the Rb protein family is not merely inactivated by HCMV but Rb protein complexes are modified in a way that promotes viral replication at multiple infection stages (66).

For MCMV, an interaction with pocket proteins has not been described. However, given the functional conservation of Rb phosphorylation for viral kinases across all human betaherpesviruses (11) and the presence of a positionally conserved LxCxE motif in M97 (20), a modulation of the Rb phosphorylation status by M97 seems likely. In contrast, the LxCxE motif of pp71 is not conserved in the distantly related MCMV proteins M82 and M83 (68) and protein levels of Rb family proteins have never been systematically analyzed in MCMV-infected cells. A better understanding of MCMV-pocket protein interactions would be an important prerequisite to investigate the *in vivo* relevance of CMV-mediated Rb regulation.

Like HCMV, aside from regulating Rb proteins directly, MCMV was found to interact with members of Rb-associated protein complexes. M97 binds LIN54, a member of the DREAM complex, functioning as the DNA-binding subunit of MuvB (20). The early viral gene product M117, but not its HCMV homolog pUL117, binds to E2F-DP dimers, leading to the activation of E2F-dependent gene transcription (69). All five tested E2F family members (E2F1-5) interact with M117 but differ in their sensitivity to M117 mutations (69). Disrupting M117-E2F interaction does not result in deficient virus growth (69), possibly because M97 and other viral factors can compensate for this loss in the control of Rb-E2F-dependent transcription.

### Stabilization of S-phase proteins by viral APC/C inhibitors.

Perhaps equally important as the *de novo* synthesis of S-phase gene products is their stabilization at the protein level. In mitosis, the activation of the APC/C, a multisubunit cullin-RING E3 ubiquitin ligase (70), triggers a reset of the cell cycle machinery by targeting its protein substrates for rapid proteasomal degradation. APC/C substrates encompass a broad range of proteins crucially involved in the control of DNA replication, nucleotide metabolism, cell cycle progression, and cell division. The APC/C achieves its exquisite substrate specificity by the alternative recruitment of two dedicated substrate adaptor subunits, CDC20 and FZR1 (also known as CDC20 homolog 1 [CDH1]), which recognize different sets of short linear degron motifs (71). In late G_1_ phase, stepwise APC/C-FZR1 inactivation by Emi1 binding and cyclin-CDK2 phosphorylation underlies the restriction point, the point of no return for cell cycle entry (72).

A growing number of viruses has been shown to target the APC/C for inactivation (73, 74). This includes HCMV which uses at least two mechanisms to inhibit the APC/C early during infection. First, the viral kinase pUL97 catalyzes the phosphorylation-dependent dissociation of FZR1 from the APC/C (75, 76), thereby mimicking the cyclin-CDK2 mediated inhibition of FZR1 at the G_1_/S border (77). Second, pUL21a binding leads to proteasomal degradation of the APC/C subunits APC1, APC4, and APC5 (76, 78). The APC1/4/5 subunits together form a structurally important “platform” that bridges the APC/C catalytic core with the substrate binding region (79). The platform subunits assemble in a highly interdependent manner, so that the lack of one subunit results in destabilization of the whole structure (80, 81). It is an open question which APC/C subunit represents the direct interaction site of pUL21a and triggers the following collapse of the APC1/4/5 platform. It is further unknown what the molecular basis for the inherent instability of pUL21a is and how it is conferred to the APC/C and to cyclin A, the other cell cycle target of pUL21a. Importantly, both cell cycle functions can be separated by the introduction of specific point mutations in the UL21a coding sequence (44, 78). This allows to investigate their individual contributions to virus growth and to the mixed G_1_/S phenotype of infected cells. In contrast to cyclin A degradation, the APC/C function of pUL21a is not essential for efficient virus replication since pUL97 acts redundantly in this pathway and secures the upregulation of APC/C substrates (44, 78, 81). Since many APC/C-FZR1 substrates are also transcriptional targets of Rb-E2F, it can be presumed that the HCMV-encoded regulators of these two major switches in gene expression act in a complementary manner to create a favorable cellular milieu for viral replication. Because pUL21a homologs are only found in primate CMVs and a possible influence of the M97 kinase on FZR1 has not yet been analyzed, it is currently unclear whether the strategy of APC/C inactivation is conserved in MCMV.

### Viral abrogation of the p53-p21 checkpoint.

The tumor suppressor p53 is a transcription factor and a main regulator of the cellular response to an array of stress stimuli (82). In an unstressed cell, p53 turns over rapidly and thus is poorly detectable due to an interaction with MDM2 that ubiquitinates p53 and thereby mediates its proteasomal degradation (83). Upon stress, such as DNA damage, p53 is phosphorylated by ATM, ATR, DNA-PK, and Chk2 kinases, leading to the destabilization of p53-MDM2 interaction, the nuclear accumulation of p53 and ultimately to the transcriptional activation of p53 target genes (84). The p53-induced expression of p21 mediates G_1_ and G_2_ cell cycle arrest by CDK1/2 inhibition in order to preserve genetic integrity (85). This arrest can be transient to allow for DNA repair or become permanent to withdraw irreversibly damaged cells from the cell cycle (86).

CMV infection elicits profound stress responses within the infected cell (87, 88), including the activation of the DNA damage checkpoint (89) and the accumulation of p53 (18, 90, 91). In the case of MCMV, gene products of the M25 locus were found to mediate this accumulation (91). M25 proteins interact with p53, leading to an increase of the p53 half-life. Concomitantly, p53 is sequestered in nuclear, dot-like accumulations by M25 and is prohibited from transcriptional activation of the p21 promoter (91). This suggests a scenario where M25 acts as a sponge for p53 molecules inhibiting their transcriptional activity.

In HCMV-infected cells, p53 is also induced and accumulates within viral replication centers in the nucleus (92). This is likely to depend on IE2, as it binds to p53, regulates its target gene activation (90, 93–95), and localizes itself to viral replication compartments (96). In addition, several other HCMV proteins were proposed as p53 regulators, such as pUL44 (97), pUL84 (94), IE1 (94, 98), and pUL28/29 and pUL38 (99). Despite the virus-induced accumulation of p53, the cellular transcriptional response is blunted as numerous p53-responsive genes are not upregulated in HCMV-infected cells. Similar to MCMV, this includes the p21 promoter which is repressed by the pUL28/29-pUL38-p53 complex (99). In addition, p21 protein was found to be actively degraded in HCMV-infected cells (100). The exact mechanism of this p21 degradation is unknown but may be related to IE2 since an interaction between p21 and IE2 was found *in vitro* (101). CKI proteins such as p21 and p27 contribute to the assembly of active cyclin D-CDK4 complexes (102–104) but act as potent inhibitors of cyclin A/E-CDK2 complexes (105). Thus, the low availability of p21 in infected cells ensures that stimulation of cyclin E expression by HCMV yields active cyclin E-CDK complexes.

The general concept of p53 upregulation, nuclear sequestration and downmodulation of its transcriptional targets, in particular p21, appears to be well conserved between HCMV and MCMV. However, based on experiments with p53 deficient cells, it was reported that p53 has a proviral role during HCMV infection, due to the dependence of some viral genes on p53 (106, 107), but an antiviral function during MCMV infection (91). The negative effects of p53 deficiency on HCMV should be interpreted with caution because they were analyzed in a single immortalized p53^–/–^ cell strain and only partially rescued by reintroduction of the p53 wild-type gene (107, 108). Also, a recent CRISPR interference and nuclease screening approach could not clarify the functional contribution of p53 to HCMV infection (109). It is therefore also conceivable that the observed differences between HCMV and MCMV are associated with divergent signatures of gene activation by p53 in human and murine cells (110). Even though p53 protein sequences are highly similar between both species, almost 50% of p53 binding sites in human cells were identified in regions not present in mice (111). More research is needed to understand the molecular details of p53 regulation and its specific relevance for CMV-infected cells.

### dNTP upregulation by viral inhibition of SAMHD1.

Replication of the CMV genome requires deoxynucleoside triphosphates (dNTPs), the building blocks of DNA. In principle, dNTPs can be synthesized *de novo* or regenerated via the salvage pathway from degradative intermediates. While the salvage pathway predominates in quiescent cells (112), the *de novo* synthesis is potently upregulated during G_1_/S transition to create sufficient supply of dNTPs for cellular DNA synthesis. The main regulator of *de novo* dNTP synthesis is the ribonucleotide reductase (RNR), which catalyzes the reduction of ribonucleotide to deoxynucleotide diphosphates (dNDPs) (113). Importantly, a properly balanced dNTP pool is required to avoid replication stress and DNA damage (114). In this context, the sterile alpha motif and histidine–aspartate domain-containing protein 1 (SAMHD1) acts as an antagonist of RNR by catalyzing dNTP hydrolysis into nucleosides and inorganic triphosphates.

SAMHD1 activity and its limitation of the cellular dNTP pool imposes a restriction on viruses that depend on a high nucleotide supply (115). This includes MCMV (116, 117) and HCMV (117–120). Interestingly, both viruses employ strategies for SAMHD1 inhibition that are partially adopted from cycling cells, where cyclin A-CDK inactivates SAMHD1 by phosphorylating a threonine residue at position 592 (human) and 603 (mouse), respectively (121–123). CMV kinases UL97 and M97 take over this function from cyclin A in infected cells and counteract the antiviral effects of SAMHD1 (116–119, 124). Furthermore, HCMV infection leads to cytoplasmic relocalization of SAMHD1 (119), interferes with the interferon-dependent transcription of SAMHD1 at early times of infection (117) and induces Cullin-Ring E3 ligase-dependent degradation of SAMHD1 protein at late times of infection (117, 120). It remains to be determined whether MCMV uses a similar combination of mechanisms to overcome SAMHD1 restriction and which viral factors contribute to the negative regulation of SAMHD1 expression.

Although less investigated in the context of CMV infection, RNR activation probably plays an equally important role as SAMHD1 inhibition for virus growth. In particular, the lack of virus-encoded enzymes for nucleotide biosynthesis, including thymidine kinase and RNR, makes CMVs dependent on the respective host factors (125). In fact, RNR inhibitor treatment negatively affects both HCMV and MCMV replication (126, 127). MCMV can induce RNR expression by IE1-mediated promoter activation (127). However, the activation of E2F target genes by CMVs and other yet-unknown mechanisms are likely to contribute to RNR expression in infected cells (128, 129).

### Viral regulation of replication licensing.

At the DNA level, cells are prepared for the onset of S phase by the formation of prereplicative complexes at the origins of replication. This process is called replication “licensing” and reinitiates after each cell division. The final and decisive step in origin licensing is the recruitment of the MCM helicase which is regulated by the MCM loading factors Cdc6 and Cdt1. Proper regulation of replication licensing is required to protect cells from aberrant origin firing, chromosomal damage, and aneuploidie (130).

Despite the fact that cyclin A degradation by pUL21a already represents a potent mechanism to inhibit cellular DNA synthesis, HCMV encodes a second mechanism to block S-phase entry, which acts at the level of replication licensing. More specifically, HCMV interferes with MCM recruitment onto chromatin even though MCM gene expression is upregulated and MCM loading factors Cdc6 and Cdt1 are readily assembled (131, 132). The viral early protein pUL117 is responsible for this effect since it antagonizes MCM chromatin accumulation (133). Interestingly, the loss of pUL117 function impairs the proper formation of viral replication compartments (134). Other possible mechanisms that may contribute to the inhibition of origin licensing during HCMV infection are the accumulation of the Cdt1 inhibitor Geminin (132) and the deregulated cyclin E expression, counteracting proper MCM loading (135). This may suggest that IE2 causes cell cycle arrest in early S phase (24, 136) by interfering with MCM loading indirectly via upregulating cyclin E. In contrast to this indirect mechanism, IE2 also interferes more directly with the MCM complex by interacting with the MCM3 acetylase (137). The fact that both pUL21a and pUL117 are required for efficient inhibition of cellular DNA replication (43, 44, 133) suggests that MCM or cyclin A inhibition alone are not sufficient to stably maintain G_1_ arrest throughout the course of HCMV infection.

In contrast to HCMV, MCMV infection does not preclude MCM helicase proteins from chromatin binding (138). This correlates with the finding that M117, the UL117 homolog of MCMV, functionally interacts with E2F-DP transcription factors but not with the proteins involved in prereplicative complex assembly (69). Although M117 contributes to the regulation of G_1_/S transition during infection (69), the antilicensing function of UL117 is apparently not conserved in MCMV.

## VIRAL SENSING OF THE G_1_/S CELL CYCLE STAGE

In addition to reprogramming the host’s cell cycle during a productive infection, murine and human CMVs are able to adapt viral gene expression and functional properties of viral proteins dependent on cellular cyclin-CDK levels.

For HCMV, the inner tegument protein pp150 (pUL32) is able to interact with host proteins after virus entry and before *de novo* viral gene expression occurs. pp150 interacts with cyclin A via an RXL motif and is subsequently phosphorylated by cyclin A-CDK. This mechanism restricts IE gene expression in S/G_2_ phases where cyclin A-dependent kinase activity is high (139). Consistently, this restriction can be extended to G_1_ phase by constitutive transgene expression of cyclin A (19) and is overcome by transient administration of chemical CDK1/2 inhibitors (140). The mechanistic details of how phosphorylation of pp150 restricts IE gene expression are unknown (141). Virion-associated pp150 has an important role in stabilizing the viral nucleocapsid by its tight association with the smallest capsid protein (pUL48.5) (142) and the formation of a net-like layer around the nucleocapsid (143). This is thought to stabilize the nucleocapsids against the high internal pressure of the large HCMV genome, possibly exceeding 20 atm (144). It would therefore be conceivable that phosphorylation of pp150 delays the release of the viral genome by impacting uncoating of the tegument or nuclear injection of the genome. Such a mechanism might contribute to the establishment of virus latency in undifferentiated cells (140). Furthermore, cyclin A sensing by pp150 cooperates with cyclin A degradation by pUL21a in preventing lytically infected cells from mitotic cell death (145).

Interestingly, animal CMVs do not contain RXL-motifs in their pp150 homologs and accordingly start gene expression independently of the cell cycle stage (47, 139). However, MCMV is able to alter functional properties of its M97 kinase dependent on cyclin A. This is enabled by the RXL-type cyclin A interaction site near the M97 N terminus, which is located in close vicinity to an NLS (20). G_0_/G_1_ cells have low to undetectable levels of cyclin A at early time points of infection, leading to exclusively nuclear M97 localization, due to its functional NLS (20). At later stages, cyclin A induction by MCMV (19) leads to cyclin A binding to the M97 RxL motif, resulting in sterical hindrance of NLS-importin interaction and cytosolic accumulation of M97-cyclin A complexes (20). As a consequence of the different M97 localization, the kinase substrate spectrum is shifted to a cytosolic pattern (20). The switch from early nuclear to late cytosolic localization of M97 may facilitate the progression of viral infection from nuclear replication to cytosolic assembly. Since MCMV carries M97 in the virion (146), it is conceivable that virion-derived M97 binds to cyclin A after infection of S/G_2_ cells. This may lead to cell cycle-dependent differences in M97-mediated protein phosphorylation in proliferating tissues. However, this possibility has yet to be explored experimentally.

## CONCLUSIONS

The inhibition of cellular DNA synthesis while at the same time maintaining an S-phase-like cellular environment represents a daunting challenge for cytomegaloviruses. Both MCMV and HCMV have therefore devoted several gene products to this task. Although HCMV cell cycle regulation has been studied for many years, the cell cycle control mechanisms of MCMV have only recently begun to be elucidated, leading to the discovery of cell cycle effector functions of M25, M97, and M117 (20, 69, 91, 116). Consistent with their similar requirements on the metabolic and replicative state of the host cell, both viruses share a number of common key targets in G_1_/S cell cycle regulation (Fig. 1). However, despite these parallels, the individual virus gene products used to exert specific cell cycle regulatory functions are remarkably divergent. This points toward convergent evolution in antagonizing critical cell cycle regulators. For example, primate CMVs have evolved pUL21a, a factor that is solely dedicated to cell cycle regulation and knocks down two important regulators of G_1_/S transition, cyclin A and APC/C (43, 44, 78). Because MCMV lacks UL21a, the respective functions need to be accomplished by other proteins. This is achieved by viral proteins such as M97, which inhibits cyclin A by cytoplasmic sequestration. Interestingly, the sequestration of cyclin A happens at the expense of the other functions of M97 (20).

In fact, the inactivation of cyclin A is central to the understanding of the cell cycle subversion strategies of HCMV and MCMV. On the one hand, because of its dual role in S phase and mitotic entry, cyclin A is the perfect viral target to simultaneously prevent cellular replication and cell division during infection (44). On the other hand, cyclin A kinase activity is also essential for the inactivation of Rb, APC/C, and SAMHD1 in cycling cells. Since viral replication depends on a high nucleotide supply and the stable expression of S-phase genes, these latter functions of cyclin A need to be substituted by CMV kinases. This explains why CMV kinases possess CDK-like activities that can compensate for endogenous CDK function of the host cell by phosphorylation of the aforementioned regulators (11, 20, 124).

Clearly, the picture of CMV cell cycle regulation is far from complete. While we have a good understanding of how MCMV and HCMV efficiently shutoff cellular DNA synthesis, we expect that more viral regulators and mechanisms will emerge that shed more light on CMV’s ability to fine-tune cellular protein levels and activities critical for the S-phase-like cellular environment. This includes, for example, the regulation of RNR (126, 127), the induction of numerous metabolic activities (147), and the regulation of B- and D-type cyclins, as well as the modulation of DREAM repressor complexes (67).

MCMV’s great advantage is the availability of animal models, thus allowing to study the consequences of its cell cycle subversion tactics in an *in vivo* context. Regarding the possible role of HCMV in oncogenesis (148), it will be interesting to analyze whether CMV cell cycle regulators functionally interfering with tumor suppressor pathways such as Rb-E2F, p53-p21, and APC/C-FZR1 can unfold their transforming potential in such experimental settings. More research on the cell cycle regulatory activities of MCMV will greatly enhance the ability to design such experiments and draw meaningful conclusions.
